# Sodium tanshinone IIA sulfonate prevents the adverse left ventricular remodelling: Focus on polymorphonuclear neutrophil‐derived granule components

**DOI:** 10.1111/jcmm.14306

**Published:** 2019-05-08

**Authors:** Shuai Mao, Shalina Taylor, Qubo Chen, Minzhou Zhang, Aleksander Hinek

**Affiliations:** ^1^ Key Discipline of Integrated Chinese and Western Medicine Second Clinical College, Guangzhou University of Chinese Medicine Guangzhou China; ^2^ Cardiovascular Institute Stanford University School of Medicine Stanford CA; ^3^ Biological Resource Center Guangdong Provincial Hospital of Chinese Medicine Guangzhou China; ^4^ Translational Medicine Hospital for Sick Children Toronto Canada

**Keywords:** left ventricular remodelling, myocardial infarction, neutrophils‐derived granule components, sodium tanshinone IIA sulfonate

## Abstract

**Aims:**

The aims of this study were to evaluate the effects of sodium tanshinone IIA sulfonate (STS) on left ventricular (LV) remodelling after for ST‐elevated myocardial infarction (STEMI).

**Methods and results:**

In this prospective, randomized clinical trial, 101 patients with the ST‐elevated MI (STEMI) and a successful reperfusion were immediately randomized to receive STS (80 mg qd for 7 days) or saline control, along with standard therapy. The primary effectiveness endpoint is the % change in LV end diastolic volumes index (%∆ LVEDVi) as measured by echocardiography from baseline to 6 months. Secondary effectiveness endpoints include 6‐month period for major adverse cardiac events (MACE), including the occurrence of recurrent myocardial infarction, death, hospitalization for heart failure and malignant arrhythmia. The 6‐month changes in %∆ LVEDVi were significantly smaller in the STS group than in the control group [−5.05% vs 3.32%; *P* < 0.001]. With respect to MACE, there was a significant difference between those who received STS (8.16%) and those patients on control (26.00%) (*P* = 0.019). Meaningfully, results of parallel tests aimed at mechanistic explanation of the reported clinical effects, revealed a significantly reduced levels of neutrophils‐derived granule components in the blood of STS treated patients.

**Conclusion:**

We found that short‐term treatment with STS reduced progressive left ventricular remodelling and subsequent better clinical outcome that could be mechanistically linked to the inhibition of the ultimate damage of infarcted myocardium by infiltrating neutrophils.

## 1 INTRODUCTION

Along with the advance of the intensive care system and revascularization therapy in particular primary percutaneous coronary intervention (PCI), the prognosis for ST‐elevation myocardial infarction (STEMI) has dramatically improved. As the number of survivors has increased, a concomitant increase in pathologic cardiac remodelling patients after MI has become a huge problem. Cardiac remodelling characterized by progressive changes in ventricle volume and impaired global function was associated with poor clinical outcome.^1^ Moreover, once remodelling is established with clinical heart failure symptoms, therapies to reverse remodelling and/or improve symptoms have limited effects. Thus, the development of novel and potentially more effective devices or pharmaceutical agents would be to prevent the remodelling from occurring in the early stage of MI setting is urgently needed.

It has been already reported that degradation of extracellular matrix (ECM), occurring after heart infarction, associates with a consequent loss of myocardium tissue integrity that allows the infarct zone to be stretched by intraventricular pressure, thereby inducing the adverse left ventricular (LV) remodelling. Compelling evidence indicates polymorphonuclear neutrophils (PMN) infiltrated in coronary plaques and the infarcted myocardium plays a pivotal role in the pathologic infarct extension and the stunning of potentially viable myocardium.^2^ In addition to producing inflammatory chemokines, neutrophils have been demonstrated mediated tissue damage by releasing matrix‐degrading enzymes and reactive oxygen species.^3^ A number of clinical trials reported the increased ratio of circulating neutrophils to lymphocyte could be recognized as a prognostic factor to predict both major adverse cardiac events and chronic LV remodelling in patients suffered from MI.^4^, ^5^ Moreover, abnormal elevations in neutrophil count in blood of patients with acute coronary syndrome undergoing primary percutaneous coronary intervention (PCI) associates with larger infarct sizes and worsening of cardiac systolic and diastolic function.^6^ Importantly, promising results obtained from preclinical models studying several pharmacological approaches aimed at interfering with the myocardial neutrophil's recruitment, confirmed their beneficial effects on reducing infarct size and the extent of the ultimate cardiac injury.^7^ Hence, suggesting that neutrophils depletion strategies could be a desirable pharmacological strategy in patients with MI at a higher risk of LV remodelling.

The cardioprotective effects of sodium tanshinone IIA sulfonate (STS), a potent pharmacological derivative compound extracted from *Salvia miltiorrhizabunge*, have been elucidated by a number of studies. Experimental preclinical data have demonstrated that administering STS therapy in rat in vivo model of ischaemia/reperfusion caused inhibition of the nuclear factor‐κB‐dependent activated accumulation of neutrophils and consequently protected the heart against myocardial reperfusion injury and reduced the infarct size.^8^ Results of the another recently published study, demonstrated that administration of STS in Beagle dogs ameliorated their ischaemia‐induced myocardial inflammation, after inhibiting NLRP3 inflammasome‐dependent JAK2‐STAT3 pathway.^9^ Moreover, administration of STS also exerted anti‐fibrotic effects on cardiac fibroblasts by downregulating generation of nicotinamide adenine dinucleotide phosphate (NADPH) oxidase‐derived reactive oxygen species.^10^ Based on these findings, it is conceivable to suggest that STS may play a protective role against post‐MI failure by limiting the neutrophils infiltrations and release of PMN‐derived granule components.

This study was designed to test whether administration of STS in patients with AMI would attenuate remodelling and reduce the occurrence of major adverse cardiac events (MACE). Importantly, the obtained results clearly demonstrated that treatment of our MI patients with this pharmacological compound, holding the unique structure of phytoestrogen, significantly alleviated the neutrophil‐mediated myocardial tissue damage and consequently reduced the net harmful post‐MI outcome.

## 2 METHODS

### 2.1 Study design

The study is a randomized, parallel‐group, controlled study comparing STS with saline control in patients who survived a STEMI after successful reperfusion. Ethics committees at Guangdong Provincial Hospital of CM reviewed and gave their approval (B2011‐41‐01). All principles of the Declaration of Helsinki were applied. This trial was registered in clinicaltrials.gov (NCT02524964).

### 2.2 Participates and intervention

The eligible patients were aged 18‐80 years, who presented within 24 hours of the onset of symptoms and signs of STEMI, defined as significant ST‐segment elevation in at least two contiguous leads according to the recommendation of the American College of Cardiology and European Society of Cardiology.^11^ The patients were not considered for enrolment if they presented with cardiogenic shock, severe heart failure (New York Heart Association class IV and need for intravenous inotropic support), sustained ventricular tachycardia or ventricular fibrillation or had return of spontaneous circulation or had identified contraindication to STS.

Consenting, eligible patients or their legally authorized representative provided written informed consent and then randomized in a ratio of 1:1 to STS or saline control. The randomization was performed with a computerized central Interactive Voice and Web Response System. All principles received guidelines‐based therapy for STEMI per the discretion of the attending cardiologist. STS was intravenously injected at 80 mg dose immediately after primary or rescue PCI and then every 24 hours for 7 days.

### 2.3 Effectiveness endpoints

The primary effectiveness endpoint is the %∆ LVEDVi measured by echocardiography from baseline to 6 months after PCI. Secondary effectiveness endpoints include composite of all‐cause death, recurrence of MI documented by symptoms and with a new rise in cardiac biomarkers and hospitalization due to heart failure events adjudicated by an independent clinical events committee using standardized definitions. Additional exploratory proteomics analyses were also performed in hope for better mechanistic explanation of the observed clinical effects of STS treatment.

### 2.4 Echocardiographic measurement of cardiac remodelling

Resting transthoracic echocardiograms will be performed before randomization and then at 6‐month follow‐up visit. Two‐dimensional echocardiographic studies were recorded and submitted to the independent, blinded laboratory for a centralized assessment of quantitative analysis after quality control. As previous reports, the selected images were digitized to obtain endocardial contours and LV cavity volumes at end diastole and end systole from apical orthogonal views including 4‐ and 2‐chamber.^12^ The modified Simpson rule was used to calculate EF value using the standard formula according to American Society of Echocardiography guideline recommendations. All measurements were measured by an experienced sonographer from 5 cardiac cycles and the mean values were considered for analysis.

### 2.5 Proteomics analysis of peripheral blood samples

The blood samples from both STS (n = 9) and saline control (n = 9) groups after 5‐day treatment were prepared and assessed the variable cytokines using a Quantibody Human Cytokine Antibody Array consisting of 440 different antibodies spotted in quadruplicate onto eight slide chips (RayBiotech, Norcross, GA, USA). The signal was acquired by fluorescence detection and quantified, and the relative expression levels of cytokines were determined by comparison between groups according to the manufacturer's instructions.

Gene ontology (GO) analysis in the set of differentially expressed factors was performed with DAVID Bioinformatics Resources 6.8 databases to investigate the associated biological process, cellular component and molecular function.^13^ Functional categories were enriched and the top 10 GO functional categories were selected. Kyoto Encyclopedia of Genes and Genomes (KEGG) database was used for pathway analysis to discover a relation that was not easily visible from the changes of individual proteins.

### 2.6 Quantification of the neutrophils‐derived granule components in peripheral blood

To further confirm the proteins response to STS treatment, plasma and serum from patients received either STS (n = 48) or saline (n = 48) before and after 5‐day treatment were measured using a sandwich enzyme‐linked immunosorbent assay (ELISA) procedure following manufacturer's protocols (R&D systems Minneapolis, MN, USA).

### 2.7 Sample size

The sample size determination is based on the primary endpoint, estimated change in LVEDVi from baseline to 6 months after STS treatment. To have >80% power and detect a treatment difference of 5% in change of LVEDVi between STS and saline control (with allocation ratio of 1:1) using a two‐sided significance level of 0.05, a minimum of 46 patients were required in each arm. A therapeutic decrease in LVEDV of 5 mL/m^2^ has been shown to be associated with favourable effects on mortality.^14^ Additional patients will be recruited to account for potential dropouts.

### 2.8 Statistical analysis

All pharmacokinetic parameters were summarized as mean ± standard deviation, median (first quartile, third quartile), percent coefficient of variation as appropriate. General linear mixed models were used to perform an intention‐to‐treat analysis that consists of all randomized patients for the effectiveness analyses. The primary and secondary endpoints were assessed using analysis of covariance with major determinants, including demographic parameters (age, sex, BMI) concomitant coronary risk factors (systemic hypertension, diabetes mellitus), peak NT‐proBNP, medication status (β‐adrenergic blockers and ACE inhibitors or ARB), that could explain the LV remodelling as covariates. The statistical tests were two tailed and conducted at the 0.05 significance level using the statistical package SPSS version 13.0 software (SPSS, Inc, Armonk, NY, USA).

## 3 RESULTS

### 3.1 Patients and baseline clinical characteristics

**Figure 1:**
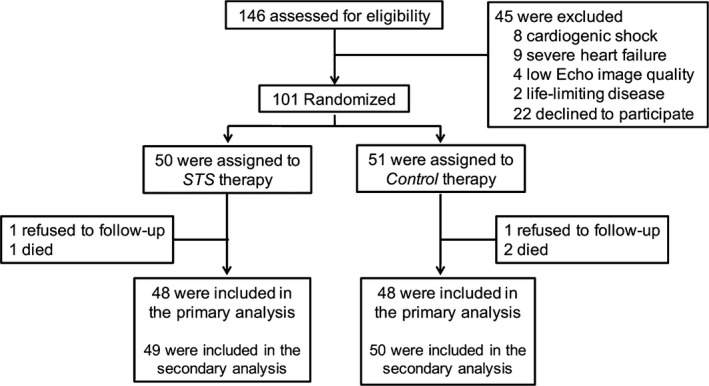
Study flow diagram. STS, sodium tanshinone IIA sulfonate

Between February 2014 and December 2016, 101 patients were randomized (50 to STS and 51 to saline control). Figure 1 illustrates study enrolment and randomization. There was one patient in the STS group and two in the control group lost to follow‐up.

Baseline demographics stratified by treatment arm are shown in Table 1. Mean ± SD patient age was 68.41 ± 9.91 years, and 50 (49.50%) were women. A total of 15 patients (14.85%) had a previous coronary heart disease, 54 (53.47%) had hypertension, 39 (38.61%) had diabetes mellitus and 35 (34.65%) had been smoking. The peak level of NT‐proBNP was 767.17 ± 76.16 pg/mL and the peak level of CK‐MB was 151.59 ± 75.89 U/L. Validated serial echocardiographic assessments at pre‐randomization and during 6‐month follow‐up were obtained for 48 patients in the STS group and 48 in the control group (Figure 1). Among these patients, there were no significant differences in LVEDVi, LVESVi or LVEF between the two groups at baseline. The LV function was well preserved, with a mean ± SD ejection fraction of 52.73%±7.92%. No differences occurred in the baseline characteristics between the two groups (Table 1).

**Table 1:** Baseline and procedural characteristics

	STS (n = 50)	Control (n = 51)	*P*
Age, y	68.19 ± 9.67	68.63 ± 10.23	0.83
Gender (Female)	24 (48.00)	26 (50.98)	0.77
Body mass index	23.79 ± 2.17	23.08 ± 2.78	0.16
Heart rate at presentation, beats/min	77.43 ± 10.39	76.26 ± 11.41	0.59
Systolic arterial pressure at presentation, mm Hg	131.75 ± 17.53	132.25 ± 17.64	0.89
Killip class at presentation	1.58 ± 0.81	1.61 ± 0.72	0.86
LVEDVi, mL/m^2^	48.53 ± 6.52	47.87 ± 7.85	0.65
LVESVi, mL/m^2^	22.41 ± 3.43	22.68 ± 4.66	0.75
EF, %	53.17 ± 8.27	52.29 ± 7.62	0.59
Previous medical history			
Coronary heart disease	7 (14.00)	8 (15.69)	0.81
Hypertension	26 (52.00)	28 (54.90)	0.77
Diabetes mellitus	20 (40.00)	19 (37.25)	0.78
Smoking	18 (36.00)	17 (33.33)	0.78
Peak NT‐proBNP, ng/L	757.95 ± 91.66	776.21 ± 113.55	0.90
Peak CK‐MB, IU/L	155.41 ± 68.19	147.84 ± 83.26	0.62
Left anterior descending artery	27 (54.00)	29 (56.86)	0.77
Infarct‐related artery TIMI flow grade 0‐1	50 (100)	51 (100)	‐
Multi‐vessel disease	14 (28.00)	13 (25.49)	0.78
Procedural success	50 (100)	51 (100)	‐
IRA stenting	50 (100)	51 (100)	‐
Number of stents	1.38 ± 0.60	1.41 ± 0.57	0.79
Stent length (mm)	24.24 ± 11.01	24.70 ± 9.92	0.83
IRA TIMI flow grade 3 after PCI	50 (100)	51 (100)	‐
Ischaemia time (min)	269.81 ± 198.77	288.81 ± 160.69	0.93
Medications			
Aspirin	47 (94.00)	48 (94.12)	1.00
clopidogrel	48 (96)	49 (96.08)	1.00
Beta blockers	38 (76.00)	36 (70.59)	0.54
ACE inhibitors/ARB	32 (64.00)	34 (66.67)	0.78
Lipid‐lowering agents	49 (98.00)	48 (94.12)	0.62
Diuretics	7 (14.00)	10 (19.61)	0.45

Data are presented as mean ± SD or median.

ACE, angiotensin‐converting enzyme; ARB, angiotensin II receptor blocker; BMI, body mass index; CK‐MB, creatine kinase‐MB; IRA, infarct‐related artery; LAD, left anterior descending artery; LVEDVi, left ventricular end diastolic volume index; LVEF, left ventricular ejection fraction; NT‐proBNP, N‐terminal pro‐brain natriuretic peptide; NYHA, New York Heart Association; PCI, percutaneous coronary intervention; TIMI, thromobolysis in myocardial infarction.

Overall, all patients displayed thrombolysis in myocardial infarction (TIMI) 3 flow within the infarct‐related artery, and there was high adherence to all post‐MI guideline‐recommended therapies. In the overall cohort, 65.35% of patients were treated with an angiotensin‐converting enzyme inhibitor or an angiotensin II receptor blocker, in comparison with 73.27% of those who received β‐adrenergic blocking agents.

### 3.2 Echocardiographic endpoints

**Figure 2:**
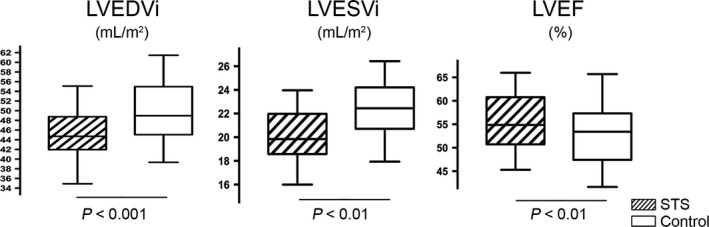
Echocardiographic changes from baseline to 6 months in LVEDVi, LVESVi and LVEF in the STS and control groups. Middle hash of the box indicates the median; 25‐75th percentiles are represented by end caps of the box; the whiskers indicate the 10th and 90th percentiles. LVEDVi, left ventricular end‐diastolic volumes index; LVESVi, left ventricular end‐systolic volume index; and LVEF, left ventricular ejection fraction

The echocardiographic measurements at 6‐month follow‐up are shown in Figure 2. The LVEDVi slightly increased in the control group, while it substantially decreased in the STS group. (mean difference in LVEDVi from 6 months to baseline assessment, 1.58 ± 1.04 vs −2.54 ± 2.48 mL/m^2^, *P* < 0.001).

The primary endpoint (%∆ LVEDVi) was significantly lower in the STS group than in the control group (−5.05 ± 4.34% and 3.32 ± 2.17%, respectively, *P* < 0.001). The covariate adjustment of STS effects on the change of LVEDVi remained significant when adjusted for fixed covariates, including age, sex, BMI, baseline heart rates and systolic arterial pressure at presentation (model 1, −4.97% relative change from pretreatment, *P* < 0.001). The effect of STS on change in LVEDVi remained significant when guideline‐based standard post‐MI medical therapies (β‐adrenergic blockers, ACE inhibitors or ARB, stains and diuretics), concomitant coronary risk factors (systemic hypertension, diabetes mellitus and peak NT‐proBNP levels), were added to model 1 (model 2, −4.95% relative change from pretreatment, *P* < 0.001).

Left ventricular end‐systolic volume index (LVESVi) slightly increased in the control group while was reduced in the STS group (1.82 ± 1.52 and −2.36 ± 1.33 mL/m^2^, respectively, *P* < 0.01). Consequently, a statistically significant difference occurred in LVEF between the two groups at the 6‐month follow‐up: a significantly higher LVEF (55.81 ± 6.98 and 49.94 ± 7.19%, respectively, *P* < 0.01) were found in the STS group compared with the control group.

### 3.3 Clinical outcome

Overall, 6‐month mortality was 2.97% (3 of 101 patients), one patient (2.04%) in the STS group and two patients (4%) in the control group. Two patients died of heart failure during the first month after index MI (one in the STS group and one in the control group), and one patient died of ischaemic stroke at 4 months after MI in the control group. Eight (16.0%) patients in the control group and three (6.12%) in the STS group had a non‐fatal recurrent MI documented by angiography and received revascularisation. One patient (2.04%) in the STS group and two patients (4.0%) in the control group had ventricular arrhythmia; 3 of 49 patients in the STS group (6.12%) and 7 of 50 patients in the control group (14.0%) had hospital readmission for congestive heart failure. Collectively, combined analysis of the most important adverse cardiovascular events (including death, reinfarction, hospitalization for heart failure and malignant arrhythmia [4 (8.16%) and 13 (26.00%) in the STS and control groups respectively]) showed an absolute reduction (*P* = 0.019). Moreover, no side effects secondary to STS treatment were observed suggesting the compound is well tolerated.

### 3.4 Effects of STS treatment on levels of biomarkers detected by proteomics

**Figure 3:**
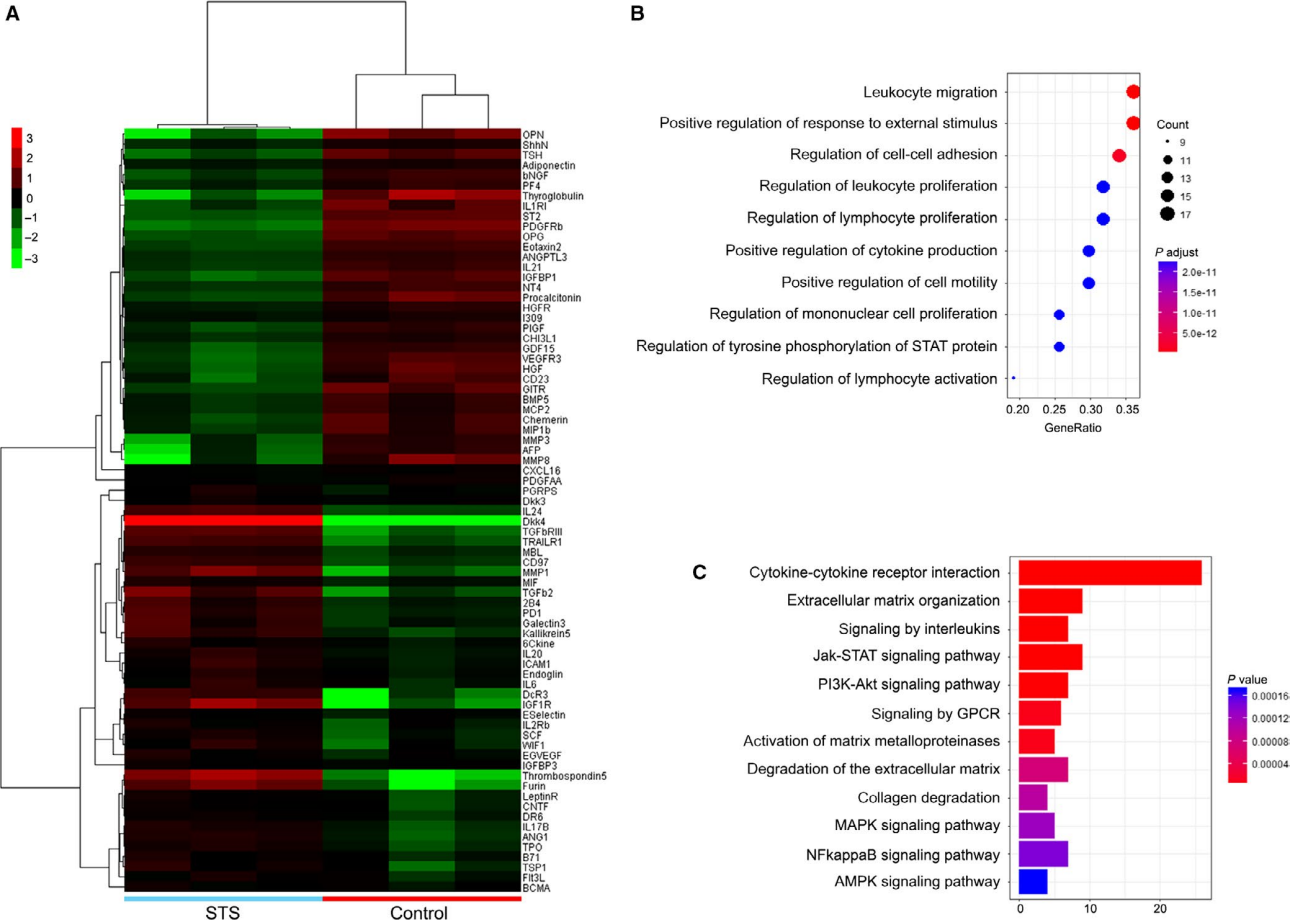
Proteomics and biosystematics analysis. A, Cluster analysis of the differentially expressed cytokines in the plasma compared between the STS patients and the control patients. B, Significant functional annotations of differentially expressed cytokines using GO analysis according to biological process ranked by enrichment score. C, KEGG pathways analysis of the differentially expressed proteins

As proteomic analysis has been already documented as a powerful tool for the identification of novel mechanisms in response to diverse pharmacologic therapies, we further investigate the mechanism underlying the anti‐remodelling effect of STS, that could be linked to alterations of numerous circulating factors, detected by antibodies microarrays in blood of patients treated either with STS or with control saline. Compared with the control patients, 75 proteins with significantly differential expression (*P* < 0.05) were identified in the STS group (Figure 3). Among them, 42 proteins were upregulated and 33 were downregulated. Cluster analysis was performed based on the differentially expressed cytokines and displayed as heat maps (Figure 3A).

Further GO analysis was carried out based on biological process, cellular component and molecular function to identify the significant regulated functional annotations of differentially expressed cytokines. Top 10 highly enriched GO terms in biological process included leucocyte migration, positive regulation of response to external stimulus, regulation of cell‐cell adhesion, regulation of leucocyte proliferation, regulation of lymphocyte proliferation and positive regulation of cytokine production (Figure 3B).

As shown in Figure 3C, applied KEGG analysis allowed us to identify particular pathways that were significantly affected by differentially expressed interleukins and their interactions with cytokine‐cytokine cellular receptors that eventually affected the cellular chemotaxis, proliferation and re‐organization of the extracellular matrix. These data encouraged the further quantitative assessment of biologically active factors that can be released from neutrophils and potentially affect myocardium.

### 3.5 Quantification of the neutrophil granules‐derived enzymes

**Figure 4:**
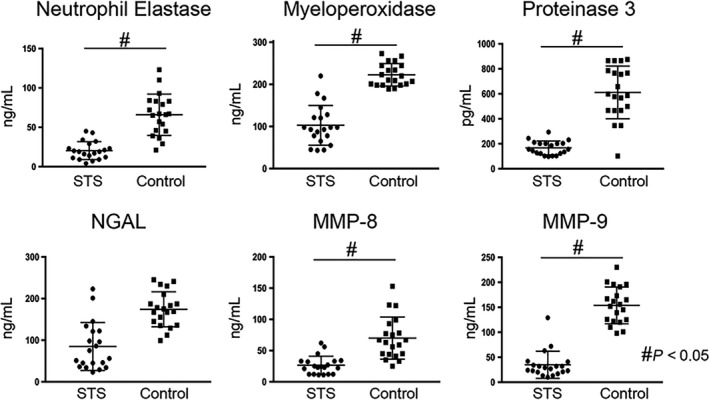
Quantification of neutrophils‐derived granule components. Levels of neutrophils‐derived granule components, including neutrophil elastase, myeloperoxidase, proteinase 3, NGAL, MMP‐8 and MMP‐9 in patients receiving STS or saline control were assessed by ELISA. NGAL, neutrophil gelatinase‐associated lipocalin. MMP, matrix metalloproteinases

As previous studies already documented that neutrophils play an essential role in tissue remodelling after several tissue injures and after MI, we now measured effects of STS treatment on blood levels of neutrophils‐derived granule components, including neutrophil elastase, myeloperoxidase, proteinase 3, neutrophil gelatinase‐associated lipocalin (NGAL), MMP‐8 and MMP‐9, in both groups of patients following MI.^15^ Peripheral blood obtained from patients just at the enrolment and after 5‐day treatment with either STS or saline were measured using ELISA with indicated antibodies. Results shown there were no differences in these neutrophils‐derived granule components between two groups at the enrolement. Interestingly, after STS treatment, the absolute count of neutrophils was not changed compared to that in the control group. However, a statistically significant difference occurred in neutrophils‐derived granule components between the two groups after 5‐day treatment: the mean values of neutrophil elastase, myeloperoxidase, proteinase 3, MMP‐8 and MMP‐9 were lower in the STS treated than in the saline control group (Figure 4). Although not significant, there was a trend towards a reduction in NGAL in patients receiving STS treatment (Figure 4).

## 4 DISCUSSION

This study demonstrates that STS treatment of MI patients who underwent the successful surgical revascularization significantly attenuates their LV remodelling and decreases in LVEDVi of ≥5% that associate with a concomitant reduction of the adverse clinical outcome. Furthermore, the degree of reduction in adverse events correlated with the magnitude of the LVEDVi decrease lending credence to a cause‐effect relationship.

The current results are consistent with the previous reports that LVEDVi is correlated with beneficial clinical outcome, and a short‐term favourable therapy effect on LVEDVi or LVEDV is associated with the improvement of longer term mortality.^16^ It has been also demonstrated that the extent of pathologic LV remodelling correlated with the risk of adverse cardiovascular events such that each 10‐mL increase in LVEDV corresponded to an adjusted hazard ratio of 1.05 (95% CI [1.01‐1.10]; *P* = 0.015) for death, MI, hospitalization for heart failure, stroke or resuscitated sudden death.^17^ Moreover, the relationship between reverse remodelling and improvement in clinical outcome was confirmed that angiotensin‐converting enzyme inhibitors (ACEI) improved survival after MI by attenuating progressive LV dilatation and dysfunction.^18^

Growing evidence from numerous studies already suggested that the initial post‐MI inflammatory oxidative stress that also occurs within the remote non‐infarcted tissues and circulated leucocytes, associates with the higher release of numerous chemokines and proteolytic enzymes that may infiltrate the adjacent infarcted myocardium, and consequently induce the partial degradation of its extracellular matrix and its pathologic remodelling that ultimately contribute to deterioration of cardiac haemodynamic. Among the other injured tissues‐derived proteases, neutrophil elastase, released in the early stages of ischaemia, degrades elastin and induces interleukin‐6 secretion to impair cardiac contractility by a nitric oxide‐dependent pathway contributing to cardiac damage. Meaningfully, it has been shown that the selective pharmacologic inhibition of neutrophil elastase protects against myocardial stunning after ischaemia/reperfusion in swine and reduce the infarct size.^19^, ^20^, ^21^, ^22^ On the other hand, it has been demonstrated that proteinase 3 exacerbate heart failure through cleaving angiotensinogen into angiotensin, activating proinflammatory factors and degrading extracellular matrix including fibronectin and collagen IV.^23^, ^24^ In addition, proteinase 3 levels in the plasma serve as a prognostic risk marker for death or rehospitalization for heart failure in post‐MI patients.^24^ Similarly, high plasma NGAL levels before PCI has been shown to independently predict all‐cause mortality for patients with MI, the underlying mechanisms involves protecting MMP‐9 from degradation.^25^, ^26^ The evidence of higher total and active MMP‐8 levels were shown in patients with LV rupture than those without rupture, indicating this neutrophil collagenase may promote infarct rupture in humans.^27^ Further studies suggested MMP‐8 degrades collagens via cleaving type I α1 and α2 chains and subsequently promotes neutrophils migration, and neutrophils depletion inhibits early collagen degradation as a consequence of the lack MMP‐8.^28^, ^29^ Infiltrating neutrophils are an early source of MMP‐9 after MI both with and without reperfusion in humans and multiple animal models.^30^ Recent reports have shown neutrophil‐derived MMP‐9 degrades extracellular matrix and promotes leucocytes infiltrate into the infarct area in the very early stage of MI.^31^ MMP‐9 deletion attenuates LV dysfunction and collagen deposition and promotes angiogenesis post‐MI in mice.^32^ Importantly, we have recently reported that STS treatment also initiate a functional transformation of myocardial fibroblasts, resulting in the inhibition of their synthesis of hard collagen type I (through inhibition of the angiotensin receptor pathway) and a concomitant increase in deposition of a new resilient elastin via the activation of PKA/CREB.^33^, ^34^ Finally, our initial finding that STS treatment of AMI patients associates with a significant reduction of neutrophil's infiltration and inhibition of their de‐granulation, strongly suggests yet another mechanism, by which this phytoestrogen may contribute to attenuation the adverse myocardial remodelling of the post‐MI heart, observed in this trial.

This is a single‐centre experience and represents a small number of patients. LV volumes were obtained by echocardiography whenever possible; while cardiac magnetic resonance may be a more precise method. However, our study population contains homogenous unselected patients with STEMI undergoing PCI, thus mirroring the real‐world scenario. Future randomized studies with a large sample size of patients with first onset of AMI with a proximal LAD occlusion are required to overcome these limitations.

## 5 CONCLUSIONS

In conclusion, our study demonstrated that addition of STS to current practice guidelines‐recommended therapies further reduces the progressive LV remodelling and improves the clinical outcome after acute MI. As, STS treatment did not reduce a total number of neutrophils detected in peripheral blood, but decreased levels of circulating chemokines and proteases, that could be released from their granules and vesicles, we suggest that the immediate daily IV treatment of MI patients with STS mitigates the early release of numerous factors that would be otherwise attracted by the degradation products of the injured myocardial matrix and ultimately exacerbate remodelling of the post‐MI heart. Therefore, at this point we might speculate that this phytomedicine might stabilize integrity of cellular membranes, thereby reducing the net level of neutrophil's exocytosis and rupture of their granules and vesicles.

## CONFLICT OF INTEREST

The authors declare that the research was conducted in the absence of any commercial or financial relationships that could be construed as a potential conflict of interest.

## AUTHORS’ CONTRIBUTIONS

SM drafted this manuscript; SM, QC and ST performed the experiments, MZZ made statistical analysis; AH made critical revision of the manuscript and contributed to the rationalization of the study. All authors read and approved the final manuscript.

## References

[1] Bhatt AS , Ambrosy AP , Velazquez EJ . Adverse remodeling and reverse remodeling after myocardial infarction. Curr Cardiol Rep. 2017;19(8):71. 28660552 10.1007/s11886-017-0876-4

[2] Carbone F , Nencioni A , Mach F , Vuilleumier N , Montecucco F . Pathophysiological role of neutrophils in acute myocardial infarction. Thromb Haemost. 2013;110(3):501‐514. 23740239 10.1160/TH13-03-0211

[3] Goldmann BU , Rudolph V , Rudolph TK , et al. Neutrophil activation precedes myocardial injury in patients with acute myocardial infarction. Free Radic Biol Med. 2009;47(1):79‐83. 19362143 10.1016/j.freeradbiomed.2009.04.004

[4] Akpek M , Kaya MG , Lam YY , et al. Relation of neutrophil/lymphocyte ratio to coronary flow to in‐hospital major adverse cardiac events in patients with ST‐elevated myocardial infarction undergoing primary coronary intervention. Am J Cardiol. 2012;110(5):621‐627. 22608360 10.1016/j.amjcard.2012.04.041

[5] Meissner J , Irfan A , Twerenbold R , et al. Use of neutrophil count in early diagnosis and risk stratification of AMI. Am J Med. 2011;124(6):534‐542. 21507368 10.1016/j.amjmed.2010.10.023

[6] Chia S , Nagurney JT , Brown DF , et al. Association of leukocyte and neutrophil counts with infarct size, left ventricular function and outcomes after percutaneous coronary intervention for ST‐elevation myocardial infarction. Am J Cardiol. 2009;103(3):333‐337. 19166685 10.1016/j.amjcard.2008.09.085

[7] Jolly SR , Kane WJ , Hook BG , Abrams GD , Kunkel SL , Lucchesi BR . Reduction of myocardial infarct size by neutrophil depletion: effect of duration of occlusion. Am Heart J. 1986;112(4):682‐690. 3766367 10.1016/0002-8703(86)90461-8

[8] Wei B , Li WW , Ji J , Hu QH , Ji H . The cardioprotective effect of sodium tanshinone IIA sulfonate and the optimizing of therapeutic time window in myocardial ischemia/reperfusion injury in rats. Atherosclerosis. 2014;235(2):318‐327. 24911635 10.1016/j.atherosclerosis.2014.05.924

[9] Hu Q , Wei B , Wei L , et al. Sodium tanshinone IIA sulfonate ameliorates ischemia‐induced myocardial inflammation and lipid accumulation in Beagle dogs through NLRP3 inflammasome. Int J Cardiol. 2015;196:183‐192. 26143630 10.1016/j.ijcard.2015.05.152

[10] Yang L , Zou XJ , Gao X , et al. Sodium tanshinone IIA sulfonate attenuates angiotensin II‐induced collagen type I expression in cardiac fibroblasts in vitro. Exp Mol Med. 2009;41(7):508‐516. 19322029 10.3858/emm.2009.41.7.056 PMC2721148

[11] Taylor J . Third universal definition of myocardial infarction. Eur Heart J. 2012;33(20):2506‐2507. 23065972 10.1093/eurheartj/ehs296

[12] Mao S , Wang L , Ouyang W , et al. Traditional Chinese medicine, Danlou tablets alleviate adverse left ventricular remodeling after myocardial infarction: results of a double‐blind, randomized, placebo‐controlled, pilot study. BMC Complement Altern Med. 2016;16(1):447. 27825334 10.1186/s12906-016-1406-4 PMC5101662

[13] da Huang W , Sherman BT , Lempicki RA . Systematic and integrative analysis of large gene lists using DAVID bioinformatics resources. Nat Protoc. 2009;4(1):44‐57. 19131956 10.1038/nprot.2008.211

[14] Kramer DG , Trikalinos TA , Kent DM , Antonopoulos GV , Konstam MA , Udelson JE . Quantitative evaluation of drug or device effects on ventricular remodeling as predictors of therapeutic effects on mortality in patients with heart failure and reduced ejection fraction: a meta‐analytic approach. J Am Coll Cardiol. 2010;56(5):392‐406. 20650361 10.1016/j.jacc.2010.05.011 PMC4523221

[15] Amulic B , Cazalet C , Hayes GL , Metzler KD , Zychlinsky A . Neutrophil function: from mechanisms to disease. Annu Rev Immunol. 2012;30:459‐489. 22224774 10.1146/annurev-immunol-020711-074942

[16] Konstam MA , Kramer DG , Patel AR , Maron MS , Udelson JE . Left ventricular remodeling in heart failure: current concepts in clinical significance and assessment. JACC Cardiovasc Imaging. 2011;4(1):98‐108. 21232712 10.1016/j.jcmg.2010.10.008

[17] Solomon SD , Skali H , Anavekar NS , et al. Changes in ventricular size and function in patients treated with valsartan, captopril, or both after myocardial infarction. Circulation. 2005;111(25):3411‐3419. 15967846 10.1161/CIRCULATIONAHA.104.508093

[18] Pfeffer MA , Braunwald E , Moyé LA , et al. Effect of captopril on mortality and morbidity in patients with left ventricular dysfunction after myocardial infarction. Results of the survival and ventricular enlargement trial. The SAVE Investigators. N Engl J Med. 1992;327(10):669‐677. 1386652 10.1056/NEJM199209033271001

[19] Bidouard JP , Duval N , Kapui Z , Herbert JM , O'Connor SE , Janiak P . SSR69071, an elastase inhibitor, reduces myocardial infarct size following ischemia‐reperfusion injury. Eur J Pharmacol. 2003;461(1):49‐52. 12568915 10.1016/s0014-2999(03)01298-6

[20] Yu X , Kennedy RH , Liu SJ . JAK2/STAT3, not ERK1/2, mediates interleukin‐6‐induced activation of inducible nitric‐oxide synthase and decrease in contractility of adult ventricular myocytes. J Biol Chem. 2003;278(18):16304‐16309. 12595539 10.1074/jbc.M212321200

[21] Jackson PL , Xu X , Wilson L , et al. Human neutrophil elastase‐mediated cleavage sites of MMP‐9 and TIMP‐1: implications to cystic fibrosis proteolytic dysfunction. Mol Med. 2010;16(5–6):159‐166. 20111696 10.2119/molmed.2009.00109 PMC2811559

[22] Akiyama D , Hara T , Yoshitomi O , Maekawa T , Cho S , Sumikawa K . Postischemic infusion of sivelestat sodium hydrate, a selective neutrophil elastase inhibitor, protects against myocardial stunning in swine. J Anesth. 2010;24(4):575‐581. 20464430 10.1007/s00540-010-0948-8

[23] Ramaha A , Patston PA . Release and degradation of angiotensin I and angiotensin II from angiotensinogen by neutrophil serine proteinases. Arch Biochem Biophys. 2002;397(1):77‐83. 11747312 10.1006/abbi.2001.2687

[24] Ng LL , Khan SQ , Narayan H , Quinn P , Squire IB , Davies JE . Proteinase 3 and prognosis of patients with acute myocardial infarction. Clin Sci (Lond). 2011;120(6):231‐238. 20942801 10.1042/CS20100366 PMC2999885

[25] Lindberg S , Pedersen SH , Mogelvang R , et al. Prognostic utility of neutrophil gelatinase‐associated lipocalin in predicting mortality and cardiovascular events in patients with ST‐segment elevation myocardial infarction treated with primary percutaneous coronary intervention. J Am Coll Cardiol. 2012;60(4):339‐345. 22813613 10.1016/j.jacc.2012.04.017

[26] Yan L , Borregaard N , Kjeldsen L , Moses MA . The high molecular weight urinary matrix metalloproteinase (MMP) activity is a complex of gelatinase B/MMP‐9 and neutrophil gelatinase‐associated lipocalin (NGAL). Modulation of MMP‐9 activity by NGAL. J Biol Chem. 2001;276(40):37258‐37265. 11486009 10.1074/jbc.M106089200

[27] van den Borne SW , Cleutjens JP , Hanemaaijer R , et al. Increased matrix metalloproteinase‐8 and ‐9 activity in patients with infarct rupture after myocardial infarction. Cardiovasc Pathol. 2009;18(1):37‐43. 18402833 10.1016/j.carpath.2007.12.012

[28] Lin M , Jackson P , Tester AM , et al. Matrix metalloproteinase‐8 facilitates neutrophil migration through the corneal stromal matrix by collagen degradation and production of the chemotactic peptide Pro‐Gly‐Pro. Am J Pathol. 2008;173(1):144‐153. 18556780 10.2353/ajpath.2008.080081 PMC2438292

[29] Harty MW , Muratore CS , Papa EF , et al. Neutrophil depletion blocks early collagen degradation in repairing cholestatic rat livers. Am J Pathol. 2010;176(3):1271‐1281. 20110408 10.2353/ajpath.2010.090527 PMC2832148

[30] Kelly D , Cockerill G , Ng LL , et al. Plasma matrix metalloproteinase‐9 and left ventricular remodelling after acute myocardial infarction in man: a prospective cohort study. Eur Heart J. 2007;28(6):711‐718. 17339265 10.1093/eurheartj/ehm003 PMC2202923

[31] Zamilpa R , Ibarra J , de Castro Bras LE , et al. Transgenic overexpression of matrix metalloproteinase‐9 in macrophages attenuates the inflammatory response and improves left ventricular function post‐myocardial infarction. J Mol Cell Cardiol. 2012;53(5):599‐608. 22884843 10.1016/j.yjmcc.2012.07.017 PMC3472138

[32] Lindsey ML , Escobar GP , Dobrucki LW , et al. Matrix metalloproteinase‐9 gene deletion facilitates angiogenesis after myocardial infarction. Am J Physiol Heart Circ Physiol. 2006;290(1):H232‐H239. 16126817 10.1152/ajpheart.00457.2005

[33] Mao S , Li W , Qa'aty N , Vincent M , Zhang M , Hinek A . Tanshinone IIA inhibits angiotensin II induced extracellular matrix remodeling in human cardiac fibroblasts–Implications for treatment of pathologic cardiac remodeling. Int J Cardiol. 2016;202:110‐117. 26408838 10.1016/j.ijcard.2015.08.191

[34] Mao S , Wang Y , Zhang M , Hinek A . Phytoestrogen, tanshinone IIA diminishes collagen deposition and stimulates new elastogenesis in cultures of human cardiac fibroblasts. Exp Cell Res. 2014;323(1):189‐197. 24525372 10.1016/j.yexcr.2014.02.001

